# Constructing diagnostic signature of serum microRNAs using machine learning for early pan-cancer detection

**DOI:** 10.1007/s12672-024-01139-1

**Published:** 2024-07-04

**Authors:** Yuyan Xu, Wei Liao, Huanwei Chen, Mingxin Pan

**Affiliations:** 1 General Surgery Center, Department of Hepatobiliary Surgery II, Zhujiang Hospital, Southern Medical University, Guangzhou, Guangdong China; 2 Department of Hepatobiliary Surgery, The First People’s Hospital of Foshan, Foshan, Guangdong China

**Keywords:** Serum, microRNAs, Pan-cancer, Machine learning, Diagnosis

## Abstract

**Background:**

Cancer is a major public health concern and the second leading cause of death worldwide. Various studies have reported the use of serum microRNAs (miRNAs) as non-invasive biomarkers for cancer detection. However, large-scale pan-cancer studies based on serum miRNAs have been relatively scarce.

**Methods:**

An optimized machine learning workflow, combining least absolute shrinkage and selection operator (LASSO) analyses, recursive feature elimination (RFE), and fourteen kinds of machine learning algorithms, was use to screen out candidate miRNAs from 2540 serum miRNAs and constructed a potent diagnostic signature (Cancer-related Serum miRNA Signatures) for pan-cancer detection, based on a serum miRNA expression dataset of 38,223 samples.

**Result:**

Cancer-related Serum miRNA Signatures performed well in pan-cancer detection with an area under curve (AUC) of 0.999, 94.51% sensitivity, and 99.49% specificity in the external validation cohort, and represented an acceptable diagnostic performance for identifying early-stage tumors. Furthermore, the ability of multi-classification of tumors by serum miRNAs in pancreatic, colorectal, and biliary tract cancers was lower than that in other cancers, which showed accuracies of 59%, 58.5%, and 28.9%, respectively, indicating that the difference in serum miRNA expression profiles among a small number of tumor subtypes was not as significant as that between cancer samples and non-cancer controls.

**Conclusion:**

We have developed a serum miRNA signature using machine learning that may be a cost-effective risk tool for pan-cancer detection. Our findings will benefit not only the predictive diagnosis of cancer but also a preventive and more personalized screening plan.

**Supplementary Information:**

The online version contains supplementary material available at 10.1007/s12672-024-01139-1.

## Introduction

Cancer is a major public health concern and a leading cause of death worldwide [1]. According to GLOBCAN statistics, there were 10 million deaths from cancer and 19.3 million new cancer cases in 2020 globally, representing a substantial economic and social distress [2]. Although life sciences have made considerable advancements in treating cancer, numerous cancer patients are first diagnosed at an advanced stage, making it one of the main reasons for the poor prognosis of patients with cancer [3]. The World Health Organization (WHO) has suggested that early detection of cancer is the most effective method to increase patient survival time, as it provides a preferable treatment window and a more optional therapeutic schedule for patients [4]. A report from Public Health England demonstrated that the five-year net survival rates for adults whose first diagnosis was colon or lung cancer within tumor stage 4 were 9.9% and 2.9%, respectively, which was substantially lower than that of patients who had their first diagnosis within tumor stage 1 (92.8% and 56.6, respectively) [5], and this is a common phenomenon in other cancers (such as liver cancer, melanoma of skin, Bladder cancer, etc.) [5]. Therefore, developing novel and effective early detection methods for cancers remains the highest priority.

There are three necessary characteristics for early cancer detection methods: low cost for widespread use, sufficient accuracy to avoid missed diagnoses, and a non-invasive or minimally invasive procedure to avoid secondary injury. Serum microRNAs (miRNAs), as biomarkers, show substantial potential as an early cancer detection method because they meet the above three fundamental characteristics [6]. miRNAs are noncoding RNA with a length of 19–25 nucleotides [7] and assist in regulating numerous biological processes, including cell differentiation, proliferation, apoptosis, and inflammation [7–9]. The expression spectra of miRNAs in tumors and normal cells are demonstrably different [10, 11]. miRNAs can be discharged into serum by exosomes and stably exist in the serum for a long period, thus reflecting the specific genetic background of the original cells [12]. These results suggest that miRNAs are appropriate cancer biomarkers. miRNA microarray technology has improved considerably after more than a decade of development. A few milliliters of blood is sufficient to detect the miRNA expression spectrum, and the cost is sufficiently low for widespread use. Recently, several studies have focused on the diagnostic value of serum miRNAs in cancer detection [13–15]. Notably, Seputra KP et al. observed that when using serum miRNA-21 to detect progressive prostate cancer, pooled sensitivity, and specificity values were 0.91 (95% confidence interval CI 0.88–0.94, I^2^ = 0%) and 0.89 (95% CI 0.85–0.92, I^2^ = 44.8%), respectively [16]. Shi Y et al. reported that serum miR-92a-1 is a novel diagnostic biomarker for colorectal cancer, and their receiver operating characteristic (ROC) analysis showed that the area under the ROC curve (AUC) was 0.914 [17]. Zhou et al. found that plasma-derived exosomal miR-15a-5p was a promising diagnostic biomarker for early detection of endometrial carcinoma, with an AUC value of 0.813 to distinguish endometrial carcinoma patients with stage I from healthy subjects [18]. The preceding study demonstrated the major utility of serum miRNA in cancer detection; however, they were limited to a specific type of cancer, and there is still an opportunity for improvement in the accuracy of these predictive models. Compared to single tumor detection models, accurate early detection models based on serum miRNA for pan-cancer are more valuable and practical, particularly in the case of a health check-up without further information. Traditional model-building methods, however, struggle to manage the exponential growth in the volume of data that must be analyzed.

Machine learning can assist in constructing an accurate diagnostic model for early cancer detection based on serum miRNAs. ‘Machine learning’ broadly refers to the process of fitting predictive models to data or identifying informative groupings within data by approximating or imitating the ability of humans to recognize patterns using computations [19]. Machine learning is particularly useful when the dataset is excessively large (many individual data points) or complex (contains a large number of features), and it can construct a more precise prediction model than traditional methods [20]. Massive amounts of serum miRNA expression data have been released in recent years, and it has become increasingly feasible to construct an accurate, cheap, and minimally invasive approach for early pan-cancer detection using machine learning. Recently, several studies attempted to construct tumor early detection models using serum miRNA and machine learning algorithms. Zhang et al. constructed a pan-cancer detection signature based on m6A target serum miRNAs using a support vector machine algorithm, with an AUC of 0.936 (95%CI 0.922—0.951) in external validation cohort [21]; Tang et al. constructed a serum diagnostic signature based on m5C-related miRNAs for pan-cancer detection, with an AUC of 0.934 in the validation cohort [22]; More recently, Wu et al. constructed a pan-cancer detection model of cell-free immune-related miRNA based on XGBoost algorithm with high accuracy in the validation set (AUC: 0.984, CI 0.980–0.989) [23]. However, limited to the machine learning analysis methods they select and the sample size in these studies, the potential of serum miRNA in pan-cancer predicting has not been fully explored. In this study, we originally optimized the machine learning workflow in constructing a pan-cancer detection model based on serum miRNA within a huge sample size of 38,223 cases and constructed an excellent pan-cancer detection signature with an AUC of 0.999 in the external validation cohort.

## Methods

**Fig. 1:**
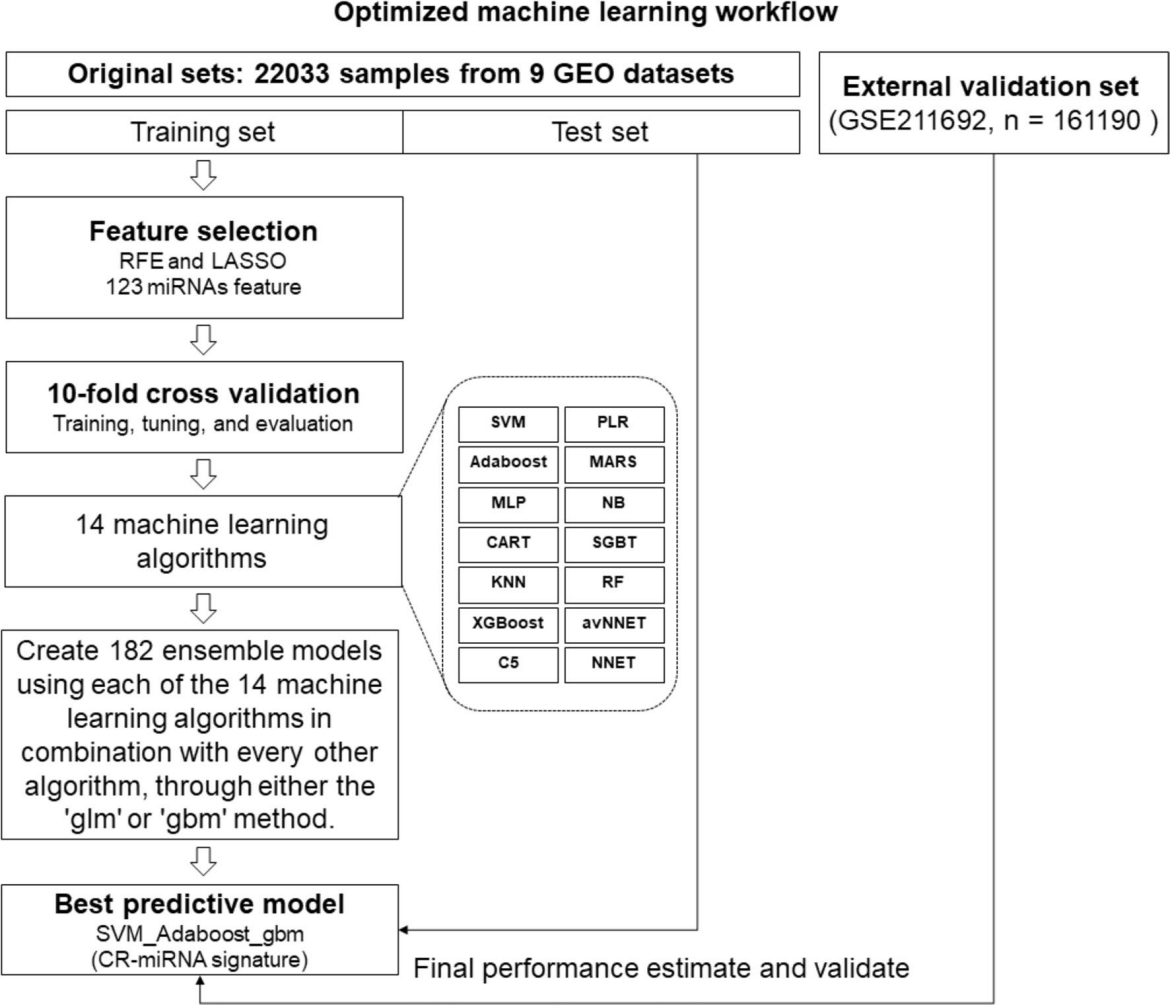
Flowchart of this study

We collected serum miRNA expression data from 38,223 samples (patients with cancer = 18,108 and non-cancer control individuals = 20,115). Based on these data, we screened a representative 123 miRNAs from 2540 serum miRNAs and constructed a cancer detection model (cancer-related miRNA signature) using an optimized machine learning workflow (Fig. 1).

### Data collection

Serum miRNA expression microarray data (normalized matrix files and corresponding clinical information) related to pan-cancer analyses were retrieved from the Gene Expression Omnibus (GEO) database. Patients with a definitive diagnosis were enrolled in this study. Patients who had received antitumor treatments, including chemotherapy, radiotherapy, targeted or immune therapy, or surgery before serum collection were excluded. Ten serum miRNA datasets were included in this study and all were obtained from the same platform, GPL21263. Nine of these datasets, namely GSE106817, GSE112264, GSE113468, GSE113740, GSE122497, GSE124158, GSE137140, GSE139031, and GSE164174, were integrated for model construction and testing. The dataset GSE211692 was used for external validation. In total, 20,115 control serum samples and 18,108 cancer serum samples were collected after eliminating duplicate samples (clinical information is shown in Supplementary Table 1). Our study included 13 cancer types: biliary tract, bladder, breast, colorectal, esophageal, gastric, hepatocellular carcinoma (HCC), intraparenchymal brain, lung, malignant bone and soft tissue, ovarian, pancreatic, and prostate. Missing values were imputed using the ‘impute’ package in R [24]. The ComBat function of the ‘sva’ package in R was used for batch correction of the different serum miRNA profiles [25].

### Data preprocessing and feature selection

First, we split the integrated data into training and test sets at a 5:5 ratio using stratified random sampling. To improve model performance and robustness, we performed Z-Score standardization [26] on the data using the preProcess function in the “caret” R package. Feature selection was then conducted using recursive feature elimination (RFE) and the least absolute shrinkage and selection operator (LASSO) algorithm. The RFE and LASSO were performed using the “caret” and “glmnet” R packages, respectively and the overlapping miRNAs screened by RFE and LASSO were extracted for further analysis. To investigate the biological processes of these miRNAs, we performed functional enrichment analysis using the miRNA Enrichment Analysis and Annotation Tool (miEAA) [27].

### Construction of binary classification diagnostic signatures

Following feature selection, 123 miRNAs were used to develop 14 types of machine learning models [26], including Support Vector Machines (SVM), multilayer perceptron (MLP), Model Averaged Neural Network (avNNET), Neural Network (NNET), Classification And Regression Tree (CART), eXtreme Gradient Boosting (XGBoost), C5 algorithm (C5), Adaptive Boosting (AdaBoost), Multivariate Adaptive Regression Spline (MARS), Naive Bayes (NB), Stochastic Gradient Boosting Tree (SGBT), Random Forest (RF), k-Nearest Neighbors (KNN), and Penalized Logistic Regression (PLR). The “caret” R package was used to construct the 14 models with tenfold cross-validation for identifying the binary classification, which can optimize model performance and avoid overfitting. The “DALEX” package was applied to evaluate model performance. Finally, to further improve the 14 diagnostic models’ performance and increase their robustness and accuracy as shown by the machine learning community [28], we ensembled pairwise combinations by setting the method as “glm” or “gbm” in the caretStack function of the “caretEnsemble” R package. Consequently, 196 serum miRNA signatures (including the model created using a single machine learning algorithm) were constructed. The serum miRNA diagnostic signature created by SVM combined with AdaBoost using the gbm method (SVM_adaboost_gbm model) showed the best performance for cancer detection and was selected for subsequent analysis. The diagnostic index, named “cancer-related miRNA signature” (CR-miRNA signature), was calculated by the predictive strength of the output of ensembles from the two machine learning classifiers. The function "predict" was applied to preprocess the dataset and quantify the prediction signature score on the training and validation cohorts.

### Construction of multiclass classification models for discriminating between different cancer types

We re-screened the representative serum miRNAs using the Elastic Net Regression Algorithm (alpha = 0.5, screening related parameters within a moderate degree) based on certain cancers within the training cohort (excluding the non-cancer controls, named as EN-training cohort) using the “glmnet” R package, and a total of 1969 serum miRNAs were screened as representative miRNAs. Based on these representative miRNAs, we re-constructed the multiple-classifying “candidate members” generated from five models, including KNN, SVM, RF, XGBoost, and MLP in the ‘tidymodels’ R package. Subsequently, we utilized a regularized linear model to combine predictions signature from ensemble members using the “stacks” R package [29], a package for model stacking that aligns with the tidymodels. Those candidate members with non-zero stacking coefficients were then fitted on the whole training set, altogether comprising a stacking Multiclass Classification model.

### Statistical analysis

Data processing was performed using R (version 4.1.3) and the R Bioconductor package. Diagnostic performance was evaluated using a ROC curve analysis, including AUC, sensitivity, specificity, and accuracy. Wilcoxon’s test was used to compare the differences nonparametrically. The 95% CIs of the evaluation metrics were estimated using the PropCI R package. Data were visualized using the ggplot2 R package. The clinical characteristics of the different datasets were analyzed using the gtsummary R package. All statistical analyses were two-sided, and statistical significance was set at p < 0.05. Principal component analysis (PCA) was performed to investigate the predictive value of the candidate serum miRNAs. To further detail the features of candidate miRNAs, we conducted an enrichment analysis based on the Gene Ontology (GO) and Kyoto Encyclopedia of Genes and Genomes (KEGG) gene sets.

## Results

### Features and functional annotation of representative serum miRNAs for constructing cancer prediction model

**Fig. 2:**
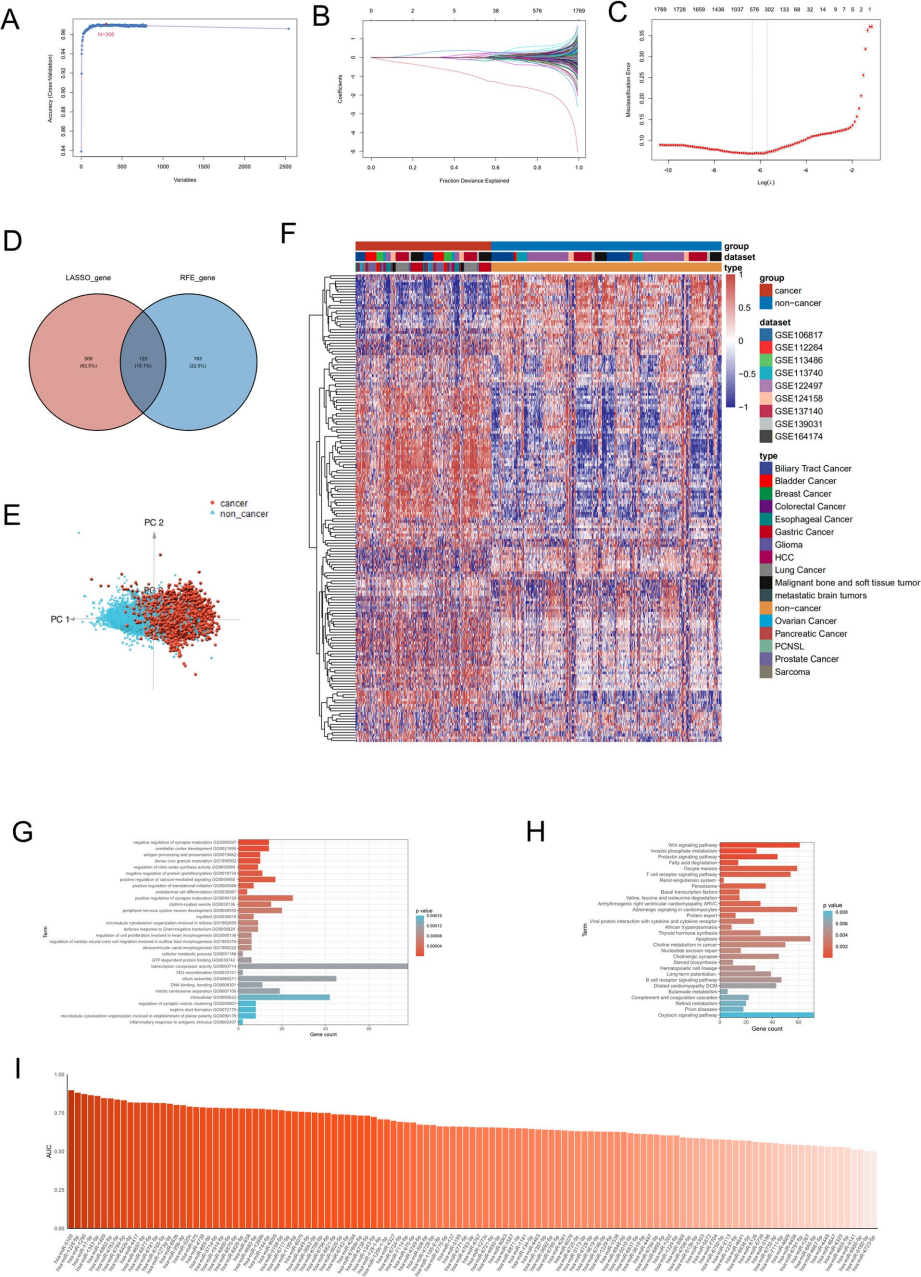
Features and functional annotation of representative serum microRNAs (miRNAs). **A** and** B**: Least absolute shrinkage and selection operator (LASSO) performed to screen the respective serum miRNAs based on the minimum criteria. **C:** Recursive feature elimination (RFE) algorithms in the training cohort. **D:** Venn diagram displaying the serum miRNAs screened by the LASSO and RFE. The miRNAs in the interaction part served as candidate miRNAs for the prediction model. **E:** Principal component analysis (PCA) of 123 candidate serum miRNAs on the training cohort. **F:** Unsupervised clustering analysis of 123 representative serum miRNAs on the training cohort. **G** and **H**: Gene Ontology (GO) (**G**) and Kyoto Encyclopedia of Genes and Genomes (KEGG) (**H**) enrichment analyses of 123 candidate serum miRNAs (the top 30 enriched terms are shown). **I**: The area under curve (AUC) of individual candidate serum miRNAs distinguishing cancer samples from non-cancer samples using the receiver operating characteristic (ROC) curve

To explore the clinical significance of serum miRNAs, we screened representative miRNAs (with independent expression features) from 2,540 serum miRNAs (Supplementary Table 2) using LASSO and RFE analyses (Fig. 2A–C) in the training cohort. The results revealed 632 and 306 specific miRNAs from the respective analyses, and 123 miRNAs were within the intersection of these two groups (Supplementary Table 3). These intersecting miRNAs served as candidates for constructing the cancer prediction model (Fig. 2D). The PCA results revealed that these 123 serum miRNAs could be used to discriminate between patients with cancer and control individuals (Fig. 2E). Unsupervised clustering analysis based on serum chip data demonstrated a significant difference in the expression of these candidate miRNAs between patients with and without cancer (Fig. 2F). These results confirmed that these miRNAs were suitable for constructing the prediction models.

GO enrichment analysis indicated that the term “transcription corepressor activity” enriched most for the candidate serum miRNAs (Fig. 2G, Supplementary Table 4). KEGG enrichment analysis showed that these miRNAs were related to multiple pathways, including the oxytocin, apoptosis, and Wnt signaling pathways (Fig. 2H, Supplementary Table 5). These results implied that these miRNAs have broad biological functions in cell regulation. Subsequently, we individually analyzed the predicted values of these miRNAs in distinguishing patients with cancer from control individuals using ROC. The results showed that serum hsa-miR-5100 had the best performance, with an AUC of 0.8976, 85.10% specificity, and 82.51% sensitivity. The performances of the other candidate miRNAs were inferior compared to those of hsa-miR-5100 (Fig. 2I, Supplementary Table 6).

### Construction of cancer-related serum miRNA signatures using machine learning algorithms

**Fig. 3:**
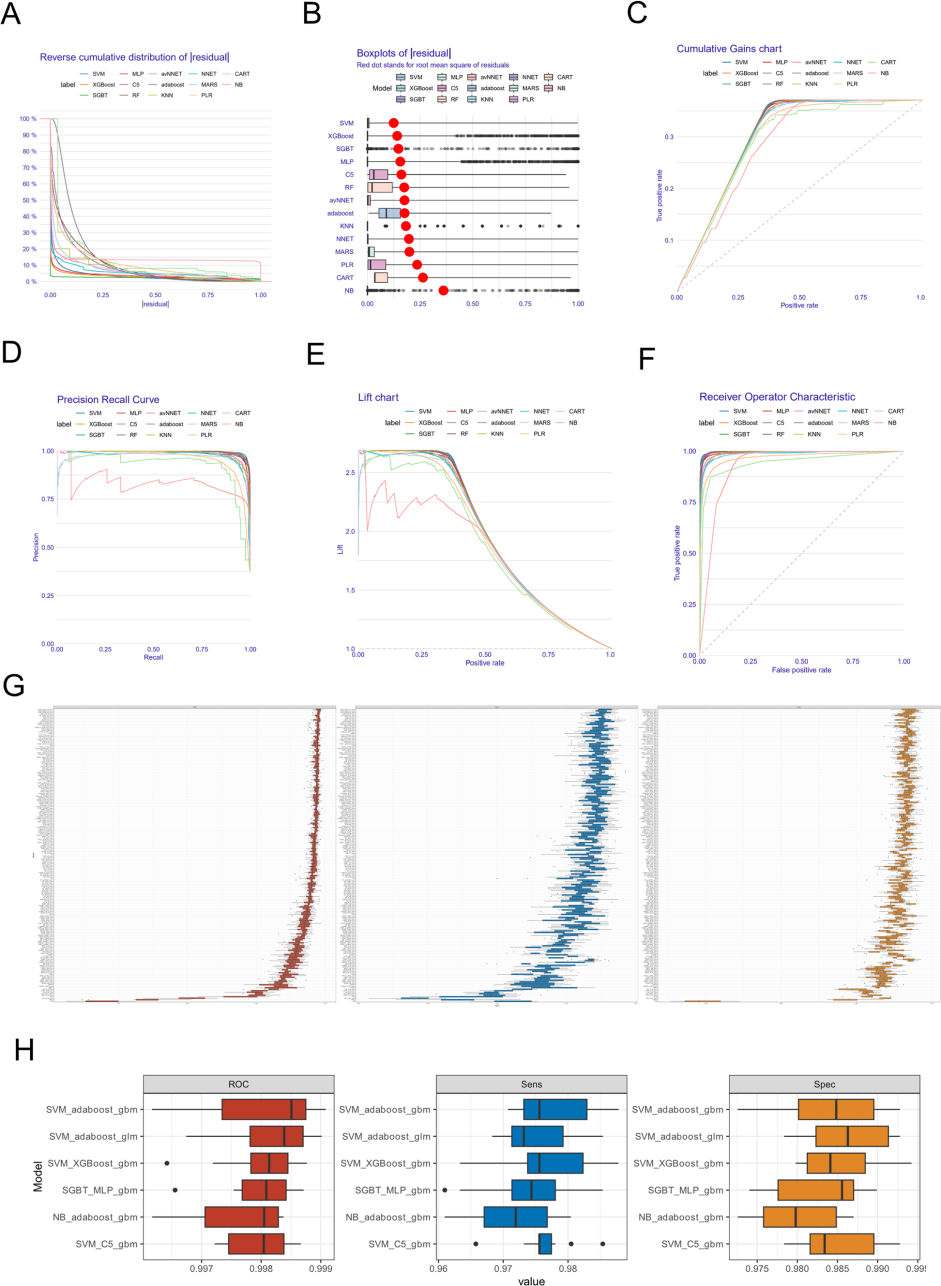
Construction of cancer-related miRNA signatures (CR-miRNA signatures). **A**–**F:** Performance indices of signatures constructed using 14 machine learning algorithms within related indices, including residual, cumulative gains, lift chart, precision recall curve, and ROC. **G:** The AUC, specificity, and sensitivity of 196 serum miRNA signatures constructed using machine learning algorithms for cancer detection. **H:** The AUC, specificity, and sensitivity of the six serum miRNA signatures with the best performance values among the 196 diagnostic models

The above analysis indicated that the candidate serum miRNAs demonstrated clinical diagnostic value for cancer detection. To construct an improved cancer detection tool, we introduced machine learning algorithms and a strategy of combining analyses to build diagnostic models to further increase detection accuracy. First, the integrated serum miRNA expression data (sequenced using the GPL21263 platform. cancer = 8187, non-cancer control = 13,156) from patients with cancer and non-cancer control individuals were randomly split into two cohorts at a ratio of 1:1 (training and test cohorts). Fourteen common machine learning algorithms, as listed in Sect. 2.3, were used independently to construct diagnostic signatures based on the training cohort. The hyperparameters for each diagnostic signature were selected according to the best ROC curve (Supplementary Fig. 1). As shown in Fig. 3A–F, comprehensively, the diagnostic signatures constructed by SVM, SGBT, XGBoost, and Adaboost performed better than the other ten signatures within the five machine learning-related indices (including residual, cumulative gains, lift chart, precision recall curve, and ROC). The median AUC of the ROC in the training cohort of the above four signatures indicated a strong performance, between 0.9972 and 0.9976 (Supplementary Table 7).

To further increase the diagnostic ability of the serum miRNA signature, we constructed new signatures via a pair-wise combination of different machine learning algorithms in two types of integrative methods (using a generalized linear model or gradient lifting trees to combine multiple base models, which were created using the “glm” or “gbm” algorithms, respectively). As a result, 196 serum miRNA signatures (including the model created by a single machine learning algorithm) were constructed, and the AUC, sensitivity, and specificity of these signatures in distinguishing cancer samples from non-cancer samples within the training cohort were obtained (Fig. 3G, Supplementary Table 8). Among the 196 diagnostic models, the serum miRNA signatures created by SVM, AdaBoost, and GBM showed the best performance for cancer detection, with a median AUC of 0.9985, median sensitivity of 0.9756, and median specificity of 0.9848 (Fig. 3H). Therefore, the subsequent analyses were all based on the “SVM_adaboost_gbm” model, which was subsequently named “cancer-related miRNA signature” (CR-miRNA signature).

### Cancer-related miRNA signature demonstrates excellent cancer detection performance

**Fig. 4:**
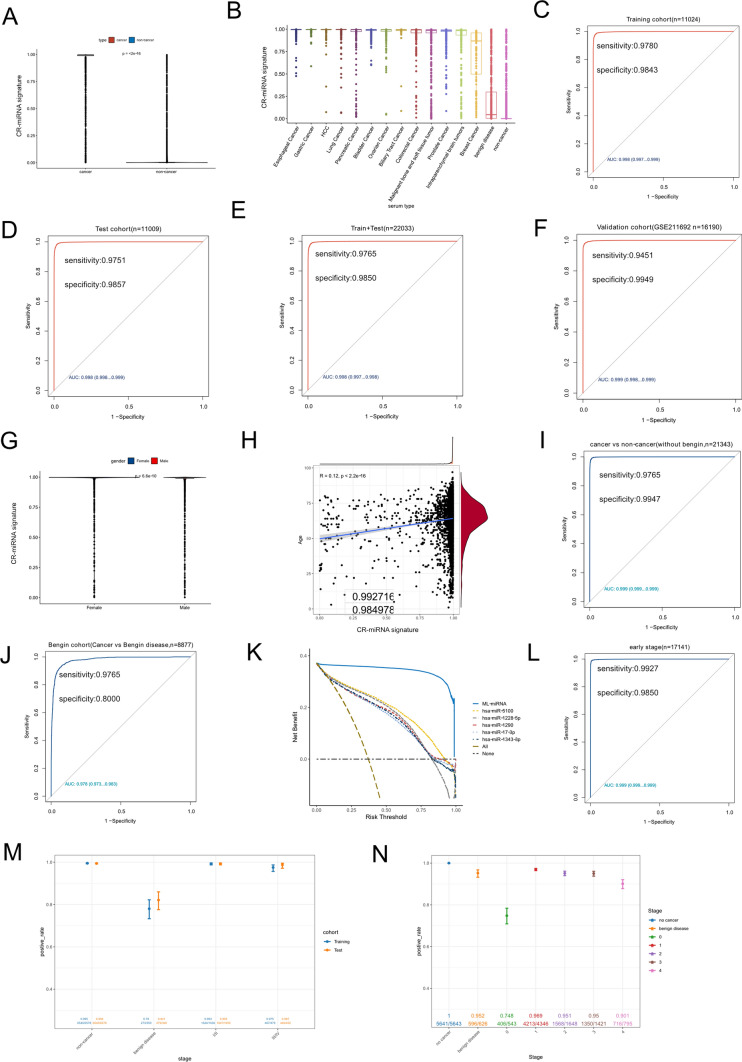
Diagnostic performance of CR-miRNA signature in cancer detection. **A: **Output strength of the CR-miRNA signature in cancer and non-cancer groups. **B:** Output strength of the CR-miRNA signature in different cancers and non-cancer groups. **C**–**F:** ROC curve showing the diagnostic performance of the CR-miRNA signature in different cohorts. **G:** Output strength of the CR-miRNA signature in samples from women and men. **H:** Correlation between output strength of the CR-miRNA signature and age of samples. **I:** ROC curve showing the diagnostic performance of the CR-miRNA signature in the cohort without cases of benign disease. **J:** ROC curve showing the diagnostic performance of the CR-miRNA signature in the cohort only containing cancers and benign disease. **K:** Difference in net benefit between CR-miRNAs and all the candidate serum miRNAs using the decision curve analysis (DCA) for a wide range of decision threshold probabilities. **L:** ROC curve showing the diagnostic performance of the CR-miRNA signature in the cohorts containing early-stage cancer samples and the non-cancer controls. M and N: Accuracy of the CR-miRNA signature in detecting cancer samples of different stages from non-cancer controls, based on the test/training (**M**) or validation cohorts (**N**)

To more comprehensively understand the diagnostic ability of the CR-miRNA signature, the output strengths of the model in different groups of the training cohort were determined. As shown in Fig. 4A, the output strength of the CR-miRNA signature in the cancer group was significantly higher than that in the non-cancer control group. Specifically, Fig. 4B depicts that the output strengths of the CR-miRNA signature in certain cancers significantly differed from those of non-cancer samples or samples from patients with benign diseases. Subsequently, the CR-miRNA signature was used to distinguish cancer samples from noncancer controls within the training, test (internal validation), and integrative cohorts (combining the training and test cohorts). The CR-miRNA signature performed strongly in these three cohorts, with an AUC > 0.998, sensitivity > 0.9751, and specificity > 0.9843 (Fig. 4C–E, Supplementary Table 9). To demonstrate the potent cancer detection ability of the CR-miRNA signature, we introduced the external validation dataset GSE211692 (from the same sequencing platform as described above). Surprisingly, the CR-miRNA signature showed excellent performance in screening cancer cases within the validation cohort, with an AUC of 0.999, sensitivity of 0.9451, and specificity of 0.9949 (Fig. 4F, Supplementary Table 9); the output strengths of the model in different groups of validation datasets were similar to those in the training cohort (Supplementary Fig. 2).

To investigate the influence of sex and age on the CR-miRNA signature, we analyzed the output strength of this model in different sexes and its correlation with age. As shown in Fig. 4G and 4H, the output strength of the model in men and women, and its correlation with age, were not clinically significant (correlation coefficient R = 0.12, p < 0.01). Some benign diseases are in a transition stage between a healthy state and cancer. To explore the influence of benign disease on the CR-miRNA signature, we used our model to screen cancer samples within two cohorts: one cohort was an integrative cohort that removed benign diseases, and the other cohort was an integrative cohort that removed healthy cases that only retained cancer samples and benign disease cases. As shown in Fig. 4I, J, when removing benign diseases, the CR-miRNA signature showed strong performance metrics in cancer detection, with an AUC of 0.999, sensitivity of 0.9765, and specificity of 0.9949. When removing healthy samples, the diagnostic ability of this model declined slightly, but it was nevertheless satisfactory, with an AUC of 0.978, sensitivity of 0.9765, and specificity of 0.8000. These results illustrate that the CR-miRNA signature can eliminate interference from benign diseases. In decision curve analyses, the CR-miRNA signature demonstrated an absolute superiority of net benefit within a wide range of decision-making threshold probabilities compared to all representative miRNAs (Fig. 4K). These analyses confirm the potential of the CR-miRNA signature for cancer detection.

A strong ability to distinguish early-stage cancers is important for cancer detection models. To explore the potential of the CR-miRNA signature for early detection, we combined a cohort containing only early-stage cancer samples with non-cancer controls. The performance of the CR-miRNA signature for early detection was satisfactory in this cohort, with an AUC of 0.999, sensitivity of 0.9927, and specificity of 0.9850 (Fig. 4L). We also compared the accuracy of cancer detection at different stages. As shown in Fig. 4M, 99.3% of early-stage cancers were correctly detected by the CR-miRNA signature in the training and test cohorts, and the non-cancer controls and advanced cancers also demonstrated a high detection accuracy (97.5%–99.6%). The CR-miRNA signature performance for distinguishing benign diseases was lower in the training and test cohorts, with accuracies of 78.0% and 82.1%, respectively. The results were similar in the validation cohort (Fig. 4N), where the accuracy of the CR-miRNA signature in identifying cancers of stages 1 and 2 were 96.9% and 95.1%, respectively. The accuracy in identifying non-cancer controls and cancers of stage 3 or stage 4 was also high, ranging from 90.1% to 99.96%. Identifying cancers of stage 0 (carcinoma in situ) had the lowest accuracy of 74.8%; nevertheless, 406 of 543 cancer samples of stage 0 were correctly distinguished.

### Prediction accuracy of serum miRNAs in defining certain cancers

**Fig. 5:**
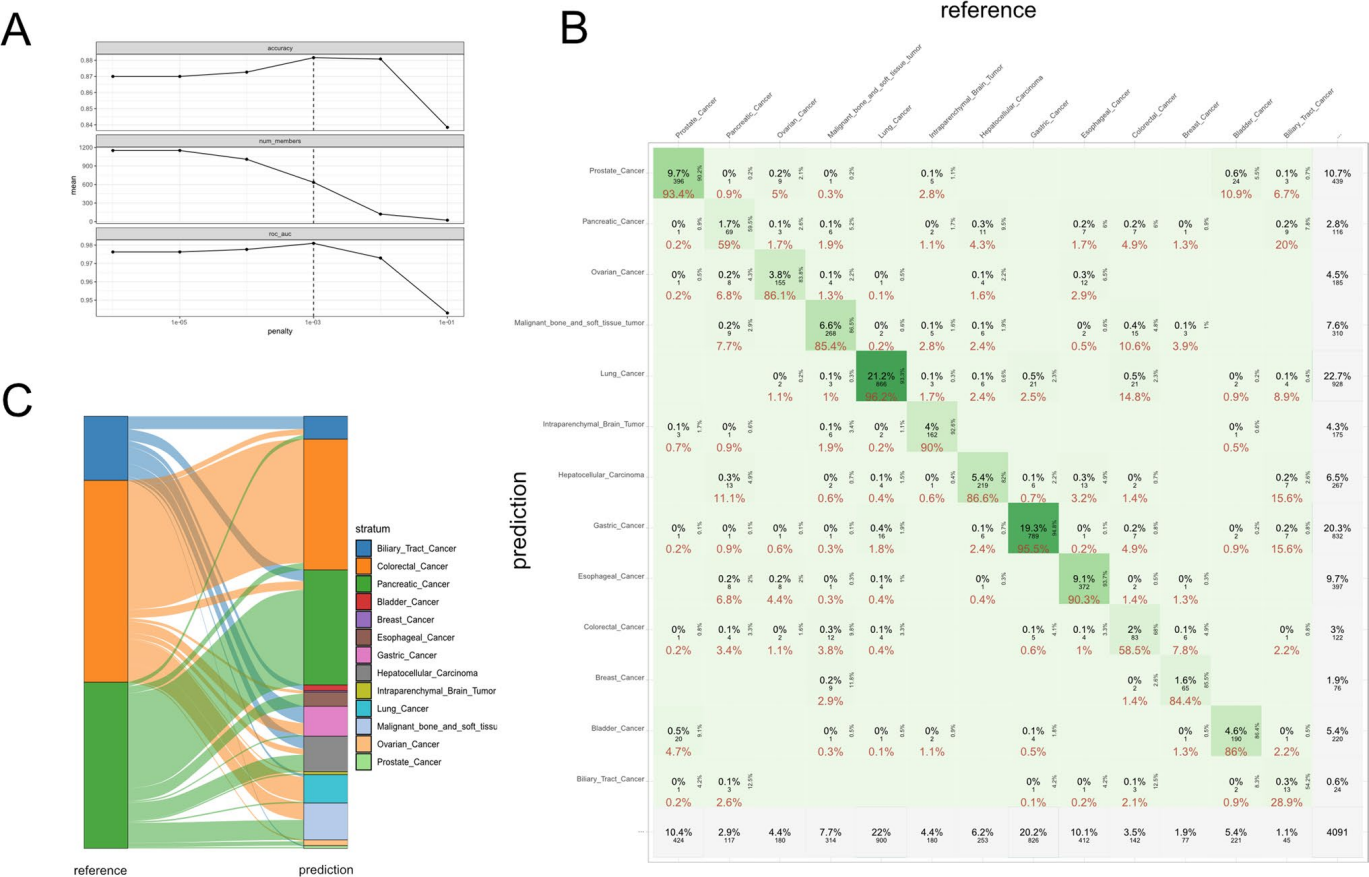
Diagnostic performance of serum miRNA signature in cancer type identification. **A**: Construction of the multiple-classifying miRNA signature using the “stacks” R package within the training cohort (excluding the non-cancer controls) according to the highest accuracy, based on 1.969 representative serum miRNAs. **B**: Diagram formulated according to the confusion matrix data generated in the cancer identifying process of multiple-classifying miRNA signature within the test cohort (excluding the non-cancer controls). The red numbers represent the percentages of the predicted cancers in certain reference cancers. The percentage in the middle of the grid is the proportion of certain predicted tumors to all samples. The digit in the middle of the grid is the number of certain predicted tumors. The gray grid displays the total amount or percentage of rows or columns. **C**: Hierarchical graph showing the flow direction of the reference cases (within pancreatic, colorectal, and biliary tract cancers) to the predicted cancers identified by the multiple-classifying miRNA signature

The CR-miRNA signature showed a strong performance in pan-cancer detection. However, the ability of serum miRNAs to predict certain cancers using machine learning remains largely unknown. The constructed CR-miRNA signature was trained using a binary classification method, which was not suitable for defining certain cancers with > 10 types. Therefore, we used the “tidymodels” and “stacks” R packages (which were suitable for constructing a prediction model for multiple classifications) to construct a multiple-classifying miRNA signature for predicting certain cancers within the training cohort (excluding the non-cancer control, named EN-training cohort), based on the above 123 candidate miRNAs. However, the performance of this signature was unsatisfactory (data not shown), considering that the number of candidate miRNAs was too small to reflect the features of thirteen cancers. Therefore, we re-screened the representative serum miRNAs by using the Elastic Net Regression Algorithm (screening related parameters within a moderate degree) based on the EN-training cohort, and a total of 1969 serum miRNAs were screened as representative miRNAs (Supplementary Table 10). Based on these representative miRNAs, we re-constructed the multiple-classifying “candidate members” generated from five model definitions, including the KNN, SVM, RF, XGBoost, and MLP algorithms in the ‘tidymodels’ R package. Subsequently, we introduced a regularized linear model to combine prediction signatures from ensemble members with the “stacks” R package within the EN-training cohort (Supplementary Fig. 3), according to the highest accuracy (Fig. 5A). We verified this signature in the test cohort (excluding non-cancer controls). In Fig. 5B (Supplementary Table 11), the multiple-classifying miRNA signature effectively distinguished most types of cancers (accuracy rate between 84.4% and 96.2%); this excluded pancreatic, colorectal, and biliary tract cancers, which showed accuracies of 59%, 58.5%, and 28.9%, respectively. As shown in Fig. 5C, the hierarchical graph presents the flow direction of the reference cases (within pancreatic, colorectal, and biliary tract cancers) to the predicted cancers identified by the multiple-classifying miRNA signature. For example, 58.5% of colorectal cancers were correctly identified, and the remaining 14.4% and 10.6% of cases were classified as malignant bone and soft tissue tumors or lung cancer, respectively; 59% of pancreatic cancers were correctly identified, and 11.1% were identified as hepatocellular carcinoma. These results indicate that the diversity of miRNA expression spectra in some tumor subtypes was not large, which affected the traceability of serum miRNAs for certain cancers.

## Discussion

In recent years, miRNAs have been investigated as promising cancer biomarkers because of the dysregulation of miRNA expression in several solid tumors [30]. The miRNAs that are aberrantly expressed in tumor cells can be partially discharged into the blood and detected, resulting in the possibility of developing a non-invasive cancer detection technique by testing the serum miRNA expression profile. Recently, several studies have found that many serum miRNAs can serve as novel cancer biomarkers in lung [31], bladder [14], and breast cancers [32]. However, most of these studies have focused on a single miRNA or a small number of serum miRNAs to detect certain cancers, and pan-cancer research is limited. Aberrant expressions of serum miRNAs in patients with cancer are complex [33]. A comprehensive analysis of the expression spectrum of serum miRNAs in patients with cancer may help identify the commonalities of cancers and improve cancer detection technology. However, compared to miRNAs in tissues, changes in miRNA expression in the blood are less readily detected because of multiple tissue sources for circulating miRNAs and multiple physiological or pathological conditions affecting miRNA quantities [34, 35]. New data processing technologies should be introduced to analyze complex and magnanimous data [36]. The development of machine learning algorithms, which can process large amounts of data and find specific features that hide in the data, has helped solve these problems.

To the best of our knowledge, this is the most extensive evaluation of serum miRNAs as biomarkers for cancer detection, and a total of 38,223 samples were incorporated into our study. The detection efficacy of the CR-miRNA signature was better than that of previous studies [21–23]. From 2540 serum miRNAs, we screened 123 candidate cancer-related miRNAs. Using an optimized machine learning analysis strategy, we constructed a CR-miRNA signature based on a training cohort. Surprisingly, this signature performed excellently for cancer detection using the expression profiles of 123 serum miRNAs. In the external validation cohort, the performance of the CR-miRNA signature in cancer detection was satisfactory. The signature’s performance in early cancer detection was also acceptable, as 96.4% of cancer samples in stages 1/2 were correctly identified. These results strongly indicate the potential of the CR-miRNA signature for cancer detection. In recent years, miRNA PCR arrays [37] that can detect the expression of miRNAs in a medium-throughput manner at a low cost have been rapidly developed. With this technique, the cancer diagnostic strategy developed in our study will be easier to promote in clinical practice, and there is a need to develop a cheap, non-invasive, and precise tool for pan-cancer detection.

In addition to exploring serum miRNA biomarkers for pan-cancer detection, we also investigated the ability of serum miRNAs to identify certain cancers. By reconstructing a serum miRNA signature suitable for multiple classifications, we found that the number of miRNAs in this signature was much higher than that in the CR-miRNA signature. A total of 1,969 miRNAs were screened for this multiple-classification miRNA signature, indicating that the serum miRNA backgrounds of different tumors were diverse. In addition, pancreatic, colorectal, and biliary tract cancers could not be accurately identified by the multiple-classification miRNA signature, with accuracies ranging from 28.9% to 59%. These results indicated that different from the remarkable difference existing in the serum miRNA profile of patients with cancer and non-cancer individuals, the diversity of miRNA expression spectra in some tumor subtypes was not significant enough, and this affects the traceability of serum miRNAs for certain cancers.

## Conclusions

In this study, we originally designed an effective machine learning workflow for constructing a pan-cancer detection tool and then used it to construct a potent predict model (named CR-miRNA signature) for pan-cancer detection with the AUC of 0.999 in the external validation cohort, based on serum miRNA expression data from a large sample size (data from 38,223 clinical samples). The CR-miRNA signature was highly effective in distinguishing early-stage cancers from healthy controls, as well as in distinguishing cancer samples from benign diseases. The development of the multiple-classifying miRNA signature for identifying certain cancers revealed the low traceability of serum miRNA in several cancers.

The findings above demonstrate the utility of the CR-miRNA signature in the early diagnosis of pan-cancer. However, there were certain limitations to our study. The pan-cancer detection method was developed using a public database; however, before we deploy it in the clinic, we need to confirm it with additional tests using clinic samples.

### Supplementary Information

Additional file1 (DOCX 3980 KB)

Additional file2 (XLSX 362 KB)

## Data Availability

The original data presented in the study are included in the article/Supplementary Material. Further inquiries including code in the present work can be directed to the corresponding authors.

## Abbreviations

AUC: Area under curve
Adaboost: Adaptive boosting
avNNET: Model averaged neural network
C5: C5 algorithm
CART: Classification and regression tree
GEO: Gene expression omnibus
HCC: Hepatocellular carcinoma
KNN: K-Nearest neighbors
LASSO: Least absolute shrinkage and selection operator
MARS: Multivariate adaptive regression spline
miRNAs: MicroRNAs
MLP: Multilayer perceptron
NB: Naive Bayes
NNET: Neural network
PCA: Principal component analysis
PLR: Penalized logistic regression
RF: Random forest
RFE: Recursive feature elimination
ROC: Receiver operating characteristic
SGBT: Stochastic gradient boosting tree
SVM: Support vector machines
XGBoost: Extreme gradient boosting
