# Supplementary material for: Constructing diagnostic signature of serum microRNAs using machine learning for early pan-cancer detection
Source: Discov Oncol. 2024 Jul 4;15:263. doi: 10.1007/s12672-024-01139-1 (PMC11224052; doi:10.1007/s12672-024-01139-1)
Supplement: Supplementary file 1 — Additional file1 (DOCX 3980 KB) [file 12672_2024_1139_MOESM1_ESM.docx]

**Supplementary figures**


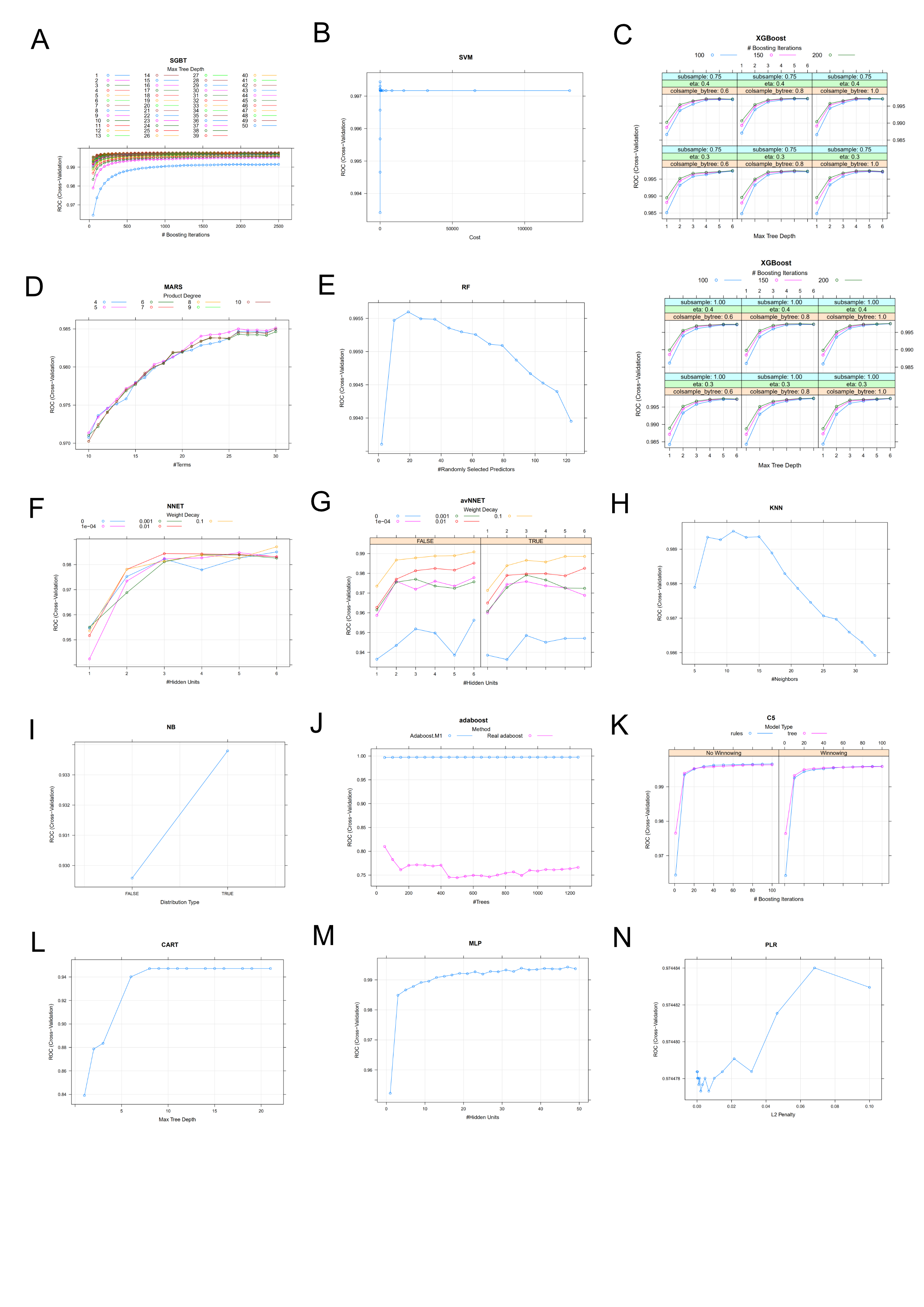


**Supplementary Figure 1.** Construction of cancer detection models using 14 machine learning algorithms. **A–N:** Hyperparameters of nine machine learning algorithms were chosen according to the best ROC curve. The fourteen evaluated algorithms included Support Vector Machines (SVM), Multi-Layer Perceptron (MLP), Model Averaged Neural Network (avNNET), Neural Network (NNET), Classification And Regression Tree (CART), eXtreme Gradient Boosting (XGBoost), C5 algorithm (C5), Adaptive Boosting (AdaBoost), Multivariate Adaptive Regression Spline (MARS), Naive Bayes (NB), Stochastic Gradient Boosting Tree (SGBT), Random Forest (RF), k-Nearest Neighbors (KNN), and Penalized Logistic Regression (PLR).


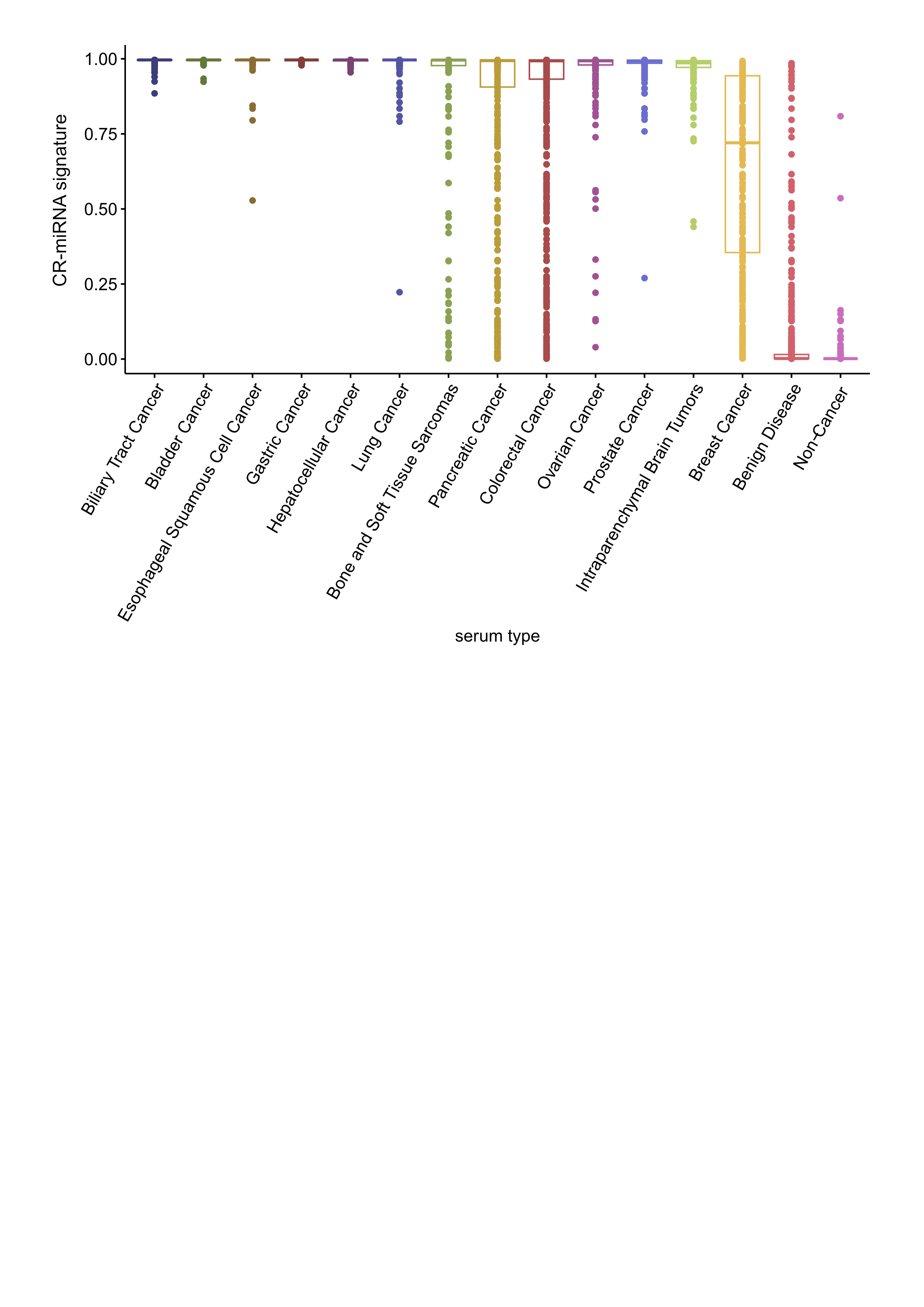


**Supplementary Figure 2.** Output strength of the CR-miRNA signature in cancer and non-cancer groups of the external validation cohort


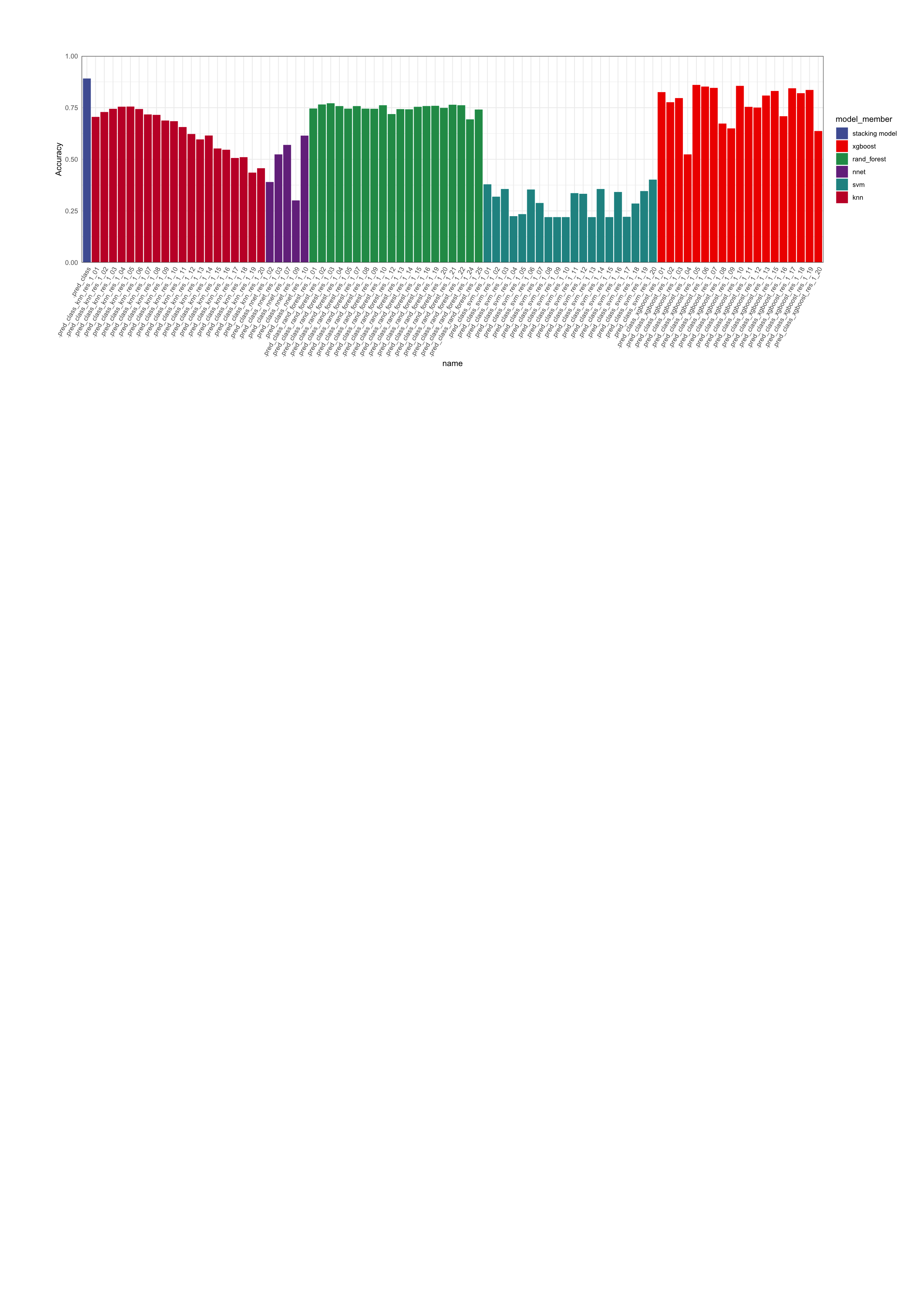


**Supplementary Figure 3.** Candidate members ensemble in the multiple-classifying miRNA signature.
